# Spontaneous Distal Migration and Protrusion of a Küntscher Intramedullary Nail from the Left Femur: A Rare Complication

**DOI:** 10.7759/cureus.6576

**Published:** 2020-01-06

**Authors:** Siddharth Jain, Hira L Nag, Prabodh Kantiwal, Devanand Hulmani

**Affiliations:** 1 Orthopedics, All India Institute of Medical Sciences, New Delhi, IND

**Keywords:** migration, protrusion küntscher nail, sinus

## Abstract

Küntscher intramedullary nail (K-nail) proximal migration in the femoral medullary canal is a common postoperative complication. But spontaneous distal migration of the K-nail across the knee joint and protrusion over the tibia till the tibial tuberosity is a complication that has very rarely been reported in the literature previously. This is the case report of a 41-year-old man who presented with a pus discharging sinus over the tibial tuberosity for the last one year. K-nail insertion was done six years ago. The underlying cause of the migration of the K-nail is subject to controversy and speculation. Infection and delayed union with shortening are some etiological possibilities. Wrong selection of K-nail size, loosely fitted nail, premature weight-bearing, and disuse osteoporosis may also be contributory factors.

## Introduction

The Küntscher cloverleaf nail (K-nail) is an intramedullary (IM) nail made of stainless steel with a slot along its longitudinal axis. In some institutions in developing countries like India, it is used for the fixation of simple transverse, short oblique, or Winquist-Hensen types I and II comminuted midshaft diaphyseal femur fractures [1]. The concept of three points fixation by long metal IM nails attached to the endosteal surface of the bone was introduced by Gerhard Küntscher et al. [2].

Küntscher IM nail migration inside the femoral medullary canal is a well-known complication. The nail can migrate proximally or distally. However, spontaneous migration of the K-nail distally across the knee joint anterior to the tibia beyond the tibial tuberosity with a pus discharging sinus is a complication that has rarely been reported in the literature previously. This was a case in which the K-nail migrated distally, perforating the distal femoral articular surface and gradually migrating across the knee joint over the tibia up to the tibial tuberosity. Selection of the wrong nail size and fixation with a faulty technique are supposed to be the commonest cause of IM nail migration [3]. However, migration of the nail has commonly been encountered after fixation of comminuted or pathological fractures, fractures associated with osteoporosis, osteogenesis imperfecta, chronically ill patients, after infection, and delayed union with shortening [4-7]. In this reported case, the cause of distal migration was uncertain because the patient did not provide previous radiographs for review.

## Case presentation

**Figure 1:**
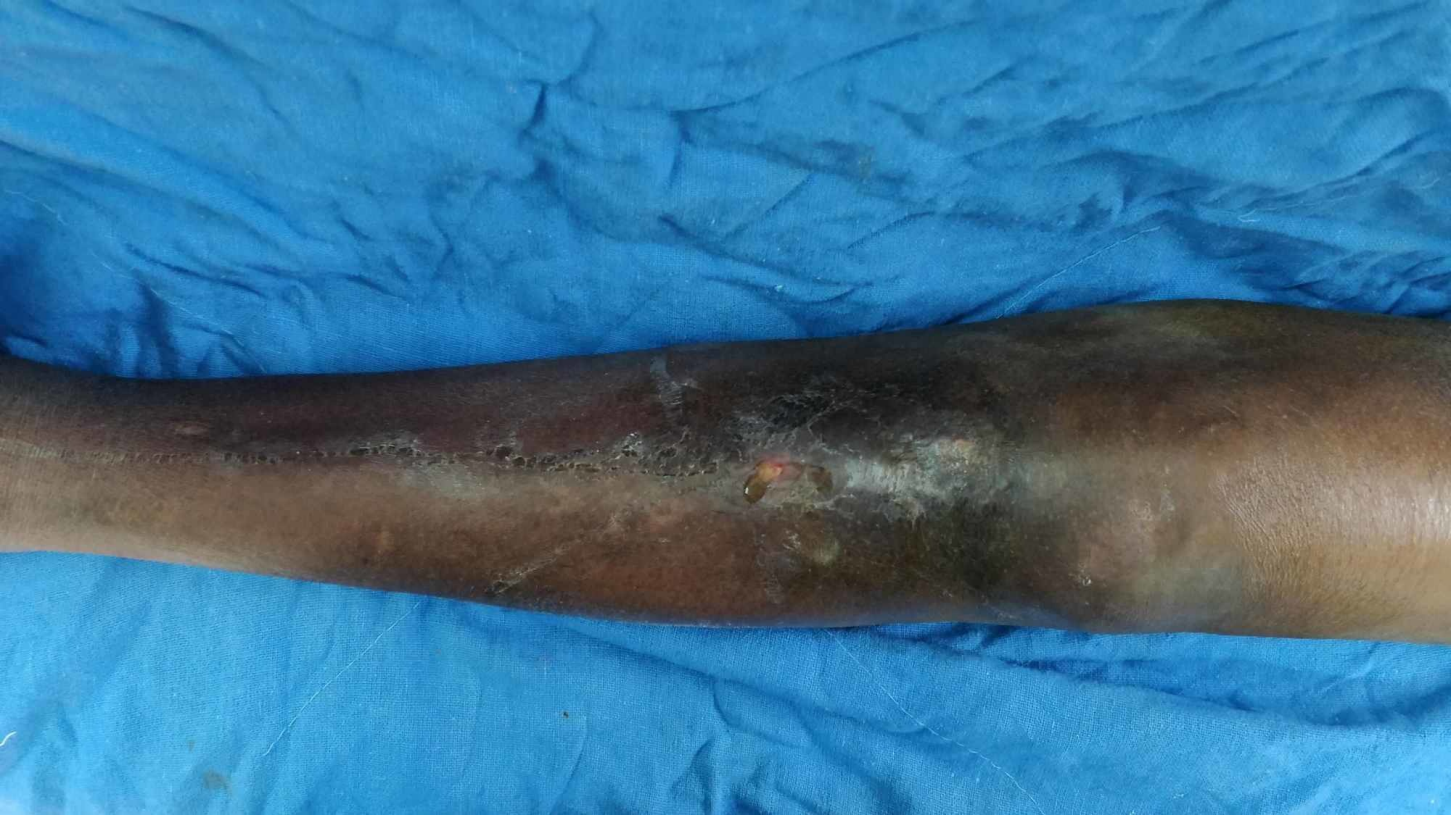
Pus-discharging sinus over the proximal leg

**Figure 2:**
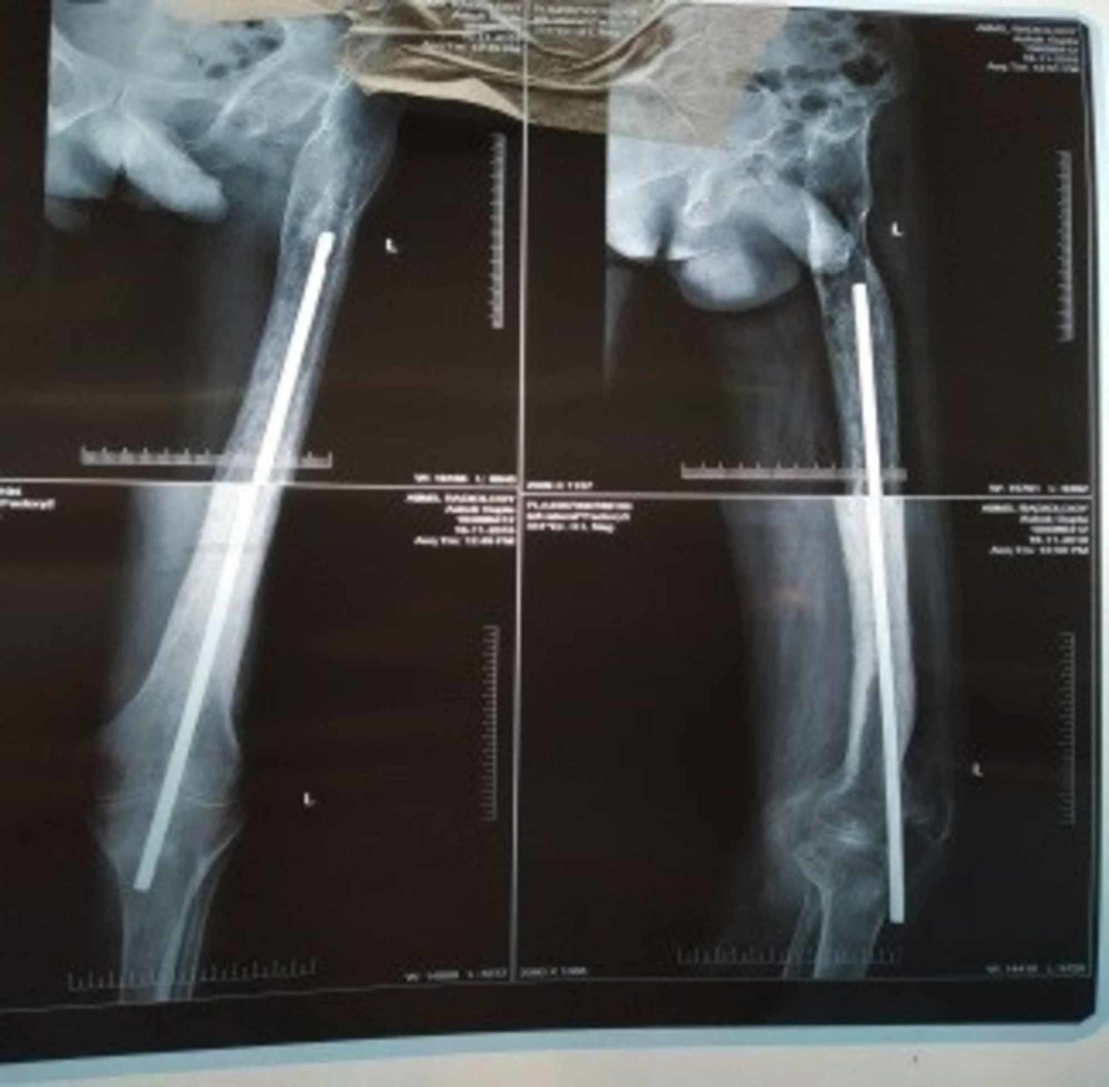
Radiograph showing distal migration of the K-nail

A 41-year-old male laborer presented at our institution with complaints of pain, swelling, and stiffness of the knee of a one-year duration and a sinus over the anterior aspect of the proximal leg. The patient was riding a vehicle involved in a road traffic accident about six years ago in which he sustained an injury to the left thigh. The thigh became painful, swollen, and deformed after the accident. The patient was unable to bear weight on the limb. There was no associated wound. He was taken to a hospital in which the attending doctor had diagnosed a fracture of the left femur after radiological investigation. He was treated by open reduction and intramedullary K-nail insertion six years ago. He had visited the same operating surgeon for follow-up care for the past five years for the complaint of pain, pus discharge, and stiffness of the knee. He was treated conservatively for the same. On examination at presentation to our institute, he was averagely built, walked with a limp, and supported with one axillary crutch. The left knee was moderately swollen, the overlying skin had blackish discoloration, with an active pus-discharging sinus over the anterior aspect of the tibial tuberosity (Figure 1). The sinus was discharging turbid purulent fluid. The knee was tender, swollen, but not fluctuant with a reduced range of motion of about 10-20 degrees. There was a healed longitudinal surgical scar on the lateral aspect of the left thigh measuring 10 cm and another healed scar over the superior aspect of the left buttock measuring 4 cm. There was no tenderness or abnormal movement at the fracture site in the left thigh. There was a limb length discrepancy of 6 cm, the left lower limb shorter than the right. No previous radiographs were available for review. Current radiographs were showing distal migration of the K-nail across the knee joint (Figure 2).

**Figure 3:**
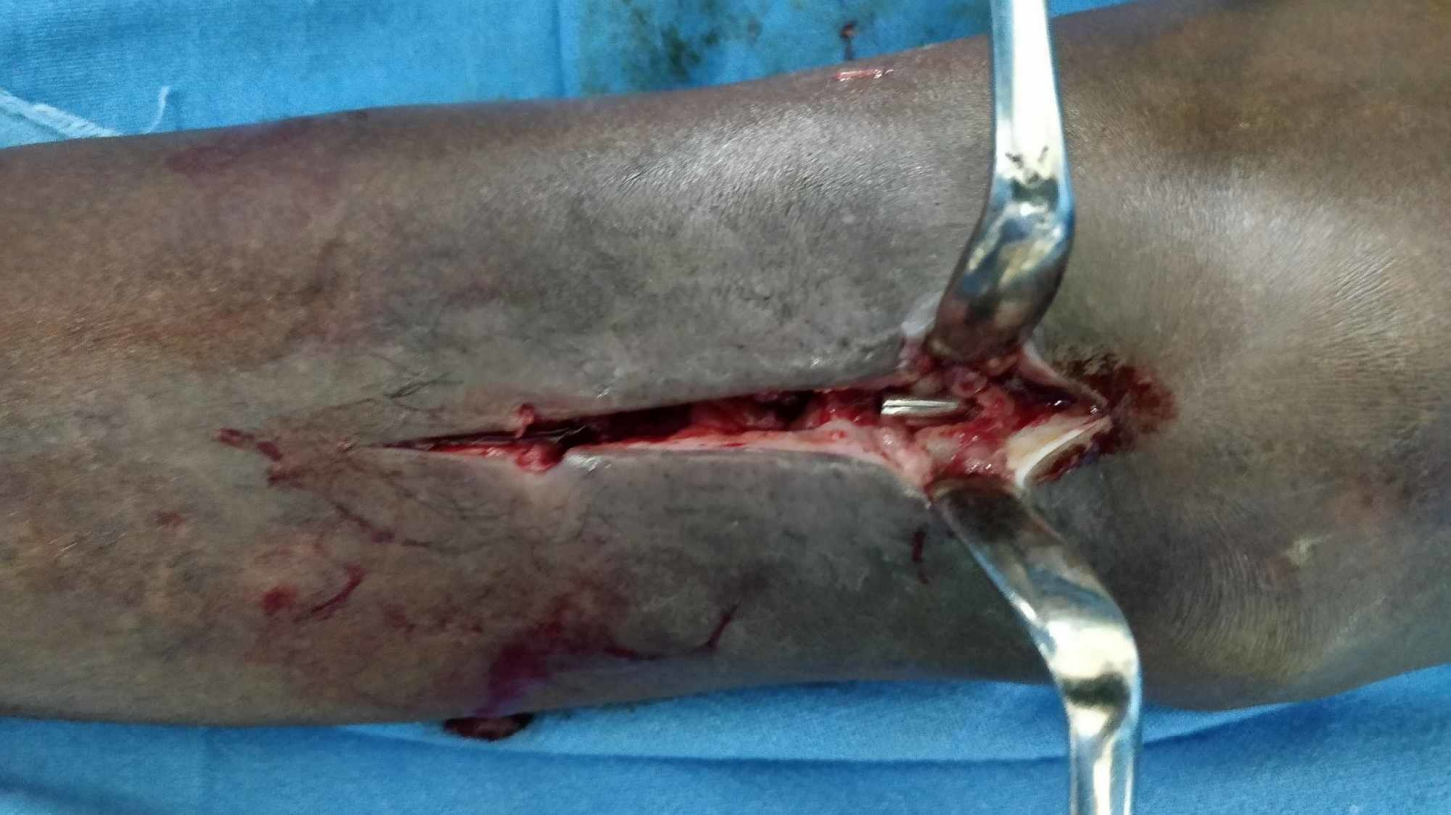
Distal end of the migrated K-nail

A 5-cm long anterior midline incision was given over the proximal left leg, distal to the inferior pole of the patella. The IM nail was identified and pulled out (Figure 3).

**Figure 4:**
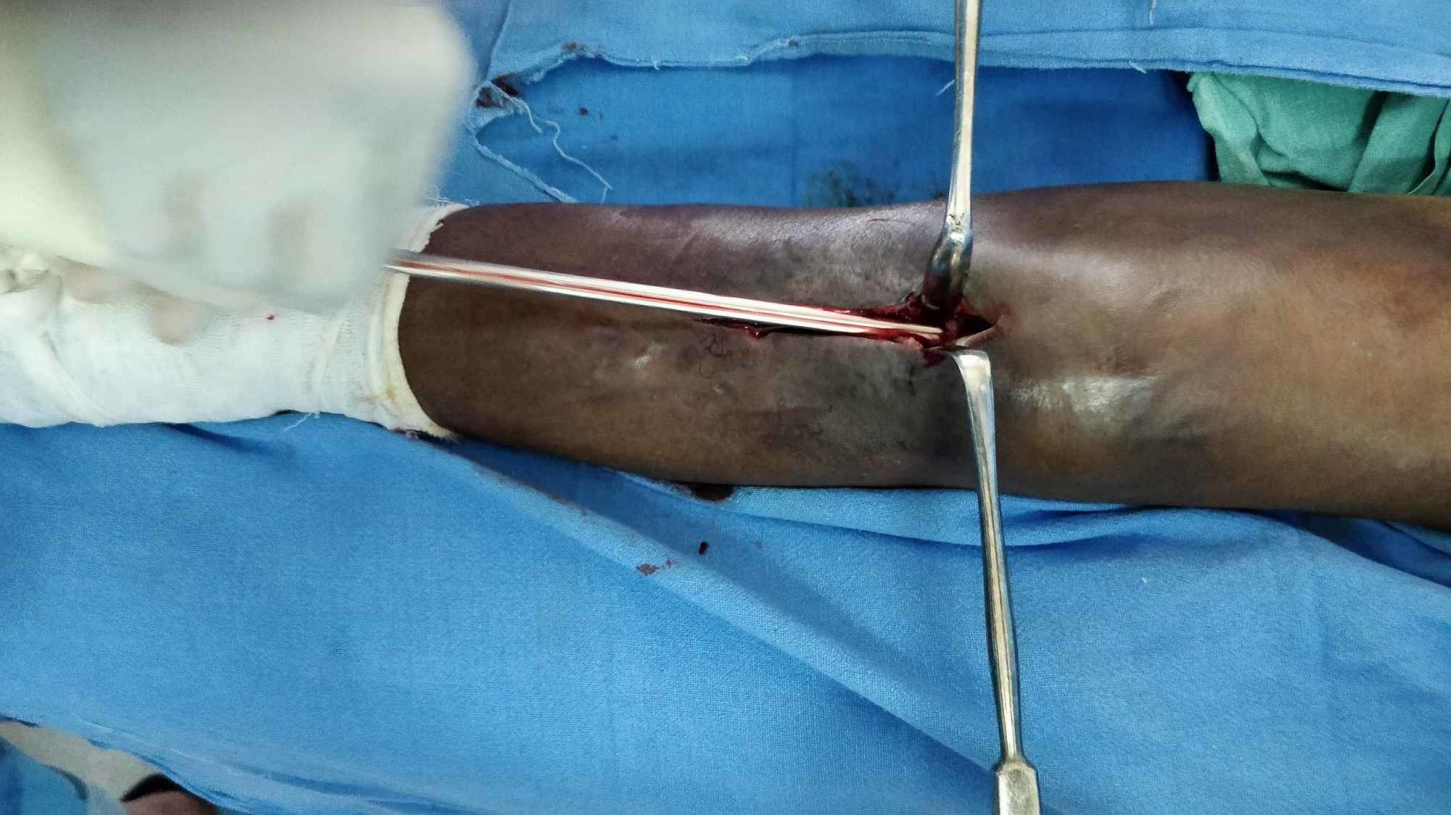
Extracted Küntscher nail

This was followed by the release of turbid purulent fluid, some of which were collected for microscopy, culture, and sensitivity. The K-nail measured 38 cm in length and 11 mm in diameter (Figure 4). The sinus was curetted and dressed. The patient was discharged on the third postoperation day and the suture was removed on the fourteenth post-operation day. Pus culture grew more than three bacterial colonies. At the one-month follow-up, the pus-discharging sinus was healed and no active discharge was present.

## Discussion

Before the introduction intramedullary nailing technique, the optimum treatment of fractures of the femur was limited to traction or a cast splint, both of which required long periods of immobilization. Küntscher nailing resulted in fast recovery and a quick return to routine activity, as the nails are a load-sharing device. However, failure to prevent migration, collapse, or rotation of the fractured fragments are some of the common problems in using the K-nail, especially in unstable fractures. These problems have been sorted out by the introduction of "locking" using interlocking bolts after the reduction of the fracture and the insertion of an appropriate size intramedullary nail. Since the interlocking nailing become so popular for the femur shaft fracture [8-9], open Küntscher nailing is no longer a common modality for the treatment of femoral shaft fractures. However, open Küntscher nailing is still commonly done in hospitals where a traction table or image intensifier is not available and when the patient has financial constraints. This patient did not present with any previous documentation and radiographs. The underlying cause of the K-nail extrusion could not be found out in this case. It was very difficult to determine the cause of migration of the K-nail from current radiological investigations. The migration and subsequent protrusion of the K-nail can be a result of the wrong selection of K-nail size, as a loosely fitted K-nail can allow repetitive movement at the fracture site. Although the 38 cm length by 11 mm diameter K-nail looked appropriate for the patient, without immediate postoperative X-rays, it was impossible to comment on this. Infection and associated delayed union with shortening can be other possible causative factors. Disuse osteoporosis and premature weight bearing can also be contributory factors. The intramedullary presence of the K-nail for a longer period of time (years) can give rise to ionization products and a foreign body reaction, which occurs between the nail and the bone and can generate a positive pressure for the migration of the nail through the weaker part of the bony cortex [10]. This complication could have been prevented if this K‑nail was removed soon after the fracture united though divided opinions have been expressed about the extraction of K-nails. After the union, extraction was not performed, and this might have led to the distal migration of the K-nail in a later stage in this case. Some surgeons prefer that unless a patient complains of local symptoms, the nail should be left for several years even after the union while some advice removal once the fracture is united and consolidated.

## Conclusions

Distal migration of the K-nail across the knee joint after femur diaphyseal fracture fixation is a rare and preventable complication. It is recommended that K-nails should be routinely removed as soon as radiological union and consolidation of the fracture is confirmed to avoid the rare postoperative complication of distal migration of the K-nail.
